# Investigation of food entry in *C. elegans* : A window into biogenic amine regulation of context dependent motility.

**DOI:** 10.17912/micropub.biology.002089

**Published:** 2026-07-07

**Authors:** James LaVilliers, James Dillon, Vincent O'Connor

**Affiliations:** 1 School of Biological Sciences, University of Southampton, Southampton, England, United Kingdom

## Abstract

Food interaction behaviours in *C. elegans* are modulated by biogenic amine signalling in response to mechanosensory cues. *C. elegans* exhibits an integrated response in which there is slowing on food contact that leads to a designated basal slowing response in the face of subsequent accumulated time on the bacterial food lawn. The discrete designation of food contact and basal slowing motility is reinforced by their selective dependence on serotonin and dopamine signalling respectively. The basal slowing is modulated by food deprivation causing a so called enhanced basal slowing (enhanced slowing). Here we present an integrated motility assay format capable of investigating the integrated response encapsulating food contact slowing and basal slowing. We compare motility in this contiguous motility assay in well-fed and food deprived worms. This approach reproduces previous findings highlighting dopaminergic control of basal slowing and serotonergic control of food entry slowing. We show that the basal slowing response seems dependent on an extended time course of dopamine signalling to sustain the slowing effect. Furthermore, we show the context dependence of food entry slowing, requiring a food deprived background to show a significant difference from the wild type. Additionally, 5-HT modulates locomotory rate during chemotaxis in a context dependent manner. The biogenic amines tyramine and octopamine do not modulate food entry slowing or basal slowing.

**Figure 1. Discrete functions of biogenic amines in food interaction behaviours:** **A.** Schematic diagram depicting assay progression from a synchronised population, to cleaning animals on an unseeded NGM plate to the two assay plate conditions used to generate observed data used in all other graphs **.** N2 vs biogenic amine mutants in a basal slowing assay showing body bends over a 20 second period in the absence of bacteria, immediately at food entry and 5 minutes post-plating onto the assay plate. For enhanced slowing measurements, worms were washed and then transferred to unseeded NGM plates for 2 hours before being assayed as described above. Letters shown in bold correspond to the graphs subsequently presented to indicate the experimental conditions used for data collection. **B.** Longitudinal assay of well-fed animals measuring body bends per 20 seconds, on food approach, food entry and then after 60 seconds for 5 minutes post food entry. Body bend comparison for food entry slowing in the green box and basal slowing in the teal box. **C.** Longitudinal assay of food deprived animals measuring body bends per 20 seconds, on food approach, food entry and then after 60 seconds for 5 minutes post food entry. **D.** N2 vs biogenic amine mutants showing body bends per 20 seconds in the absence of food. **E.** N2 vs biogenic amine mutants showing body bends per 20 seconds prior to food entry in well-fed and food deprived conditions.**** All data shown as mean ±SEM. Statistics performed: 2-Way ANOVA with Dunnett's multiple comparison with significance as ns p>0.05, * p≤0.05, ** p≤0.01, *** p≤0.001, **** p≤0.0001. Except **D** where a 1-Way ANOVA with Dunnett's multiple comparison was performed. **A:** Created in BioRender. Lamb, J. (2026) https://BioRender.com/7ugsmk4

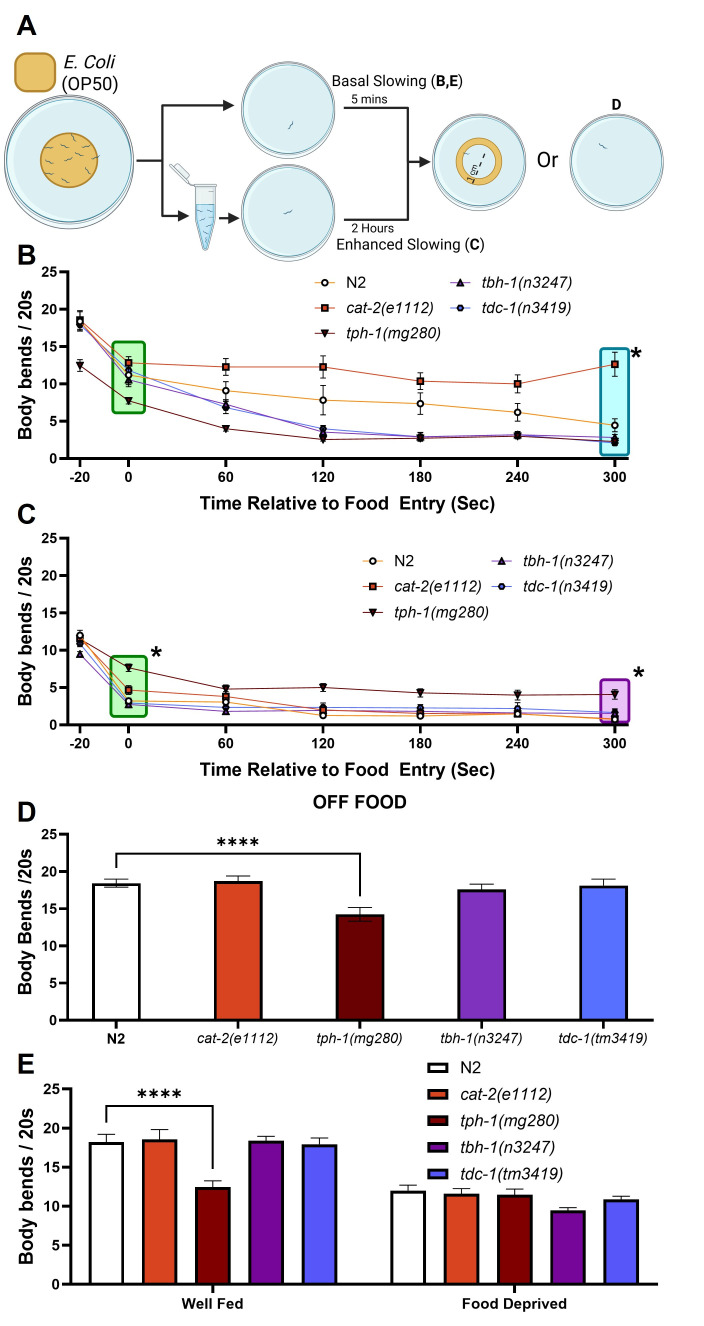



## Description

*C. elegans* exhibit a well characterised chemotaxis towards bacteria before entering and utilising this food source. Their response to encountering food has been characterised by two behaviours namely food entry slowing and the subsequent basal slowing after entering food. Previous assay formats designed to score these behaviours use time windows that score them in isolation and showed they are controlled by separate biogenic amines, serotonin (5-HT) (Iwanir et al., 2016) and dopamine (DA) (Sawin et al., 2000). In reality the two behaviours represent an integrated response which allows *C. elegans* to move towards food and exploit preferred sources when they move onto them. This is well represented by the integrated motility assay in which we combine protocols and time windows from established methods (Sawin et al., 2000 and Iwanir et al., 2016). This allowed us to define the distinct stages of changes in motility as worms enter food after chemotaxis and remain on food after this initial entry are observable.

Food entry slowing has been shown to be a 5-HT dependent behaviour in food deprived animals in which the animal performs an abrupt reduction in locomotory rate immediately after (3-5 sec) contact with the bacterial lawn (Iwanir et al., 2016). The basal slowing response is a DA dependent reduction in the locomotory rate of *C. elegans* recorded five minutes after being on food (Sawin et al., 2000). These behaviours define instantaneous and acute interaction with food, so we performed a food entry assay (Fig 1A) in both well-fed and food deprived worms. This allowed quantification of motility across food entry slowing to the basal slowing response in distinct food states. Performing this comparison is important as food deprived worms have been shown to enhance the slowing effect after 5 minutes post food entry in a 5-HT dependent manner(Sawin et al., 2000) showing the possibility for additional modulation of locomotory behaviours based on prior food availability. The role of other biogenic amines in modulating these behaviours have not been reported. This is surprising as both octopamine (OA) (Alkema et al., 2005), and tyramine (TA) (Pirri et al., 2009) interplay with other biogenic aminesin other *C. elegans* behaviours (Suo et al., 2009). Indeed, OA and TA receptors are found throughout the *C. elegans* nervous system including neurons that are core to both food entry slowing and basal slowing (Rex et al., 2004). Thus, further understanding of these behaviours is important to understand distinct biogenic amine roles in how *C. elegans* senses and responds to environmental stimuli such as food.

We performed longitudinal video analysis of well-fed and food deprived animals encompassing wild type and transmitter deficient mutant worms (Fig 1A). The assay format used here is a recreation of the original basal slowing assay format (Sawin et al., 2000) with additional observation points to capture three key behaviours. This makes our assay format a combination of those performed previously, using the physical layout of the basal slowing assay plate (Sawin et al., 2000) and continuous observations used for food entry slowing (Iwanir et al., 2016). Comparisons of locomotion between N2 and biogenic amine mutants was performed across chemotaxis, food entry slowing and the basal slowing response. In this way the assay provides an avenue to investigate integrated motility behaviours.

To ensure we are reproducing the food entry slowing and basal slowing response behaviours, we measured animals locomotory rate immediately after food entry, after food entry up to 5 minutes (Fig 1B, C) and on unseeded plates (Fig 1D). We observed in well-fed animals (Fig 1B) the DA dependence of a gradual slowdown in transition from the initial food entry slowing to a dwell state which reproduces previous findings on the basal slowing response (Sawin et al., 2000). The DA deficient *cat-2 ( e1112 )* mutants phenocopied the N2 prior to food entry, immediately post food entry but significantly deviated from the N2 at five minutes post food entry (Fig 1B, teal box). Additionally, in systematically comparing locomotory rates in well-fed animals we show DA was the only biogenic amine required to elicit the basal slowing response since the difference between the wild type and the 5-HT ( *tph-1 ( mg280 )* ), OA ( *tbh-1 ( n3247 )* ) and OA/TA ( *tdc-1 ( n3419 )* ) deficient mutants at five minutes post food entry was not significantly different. These results suggest DA plays no role in modulating motility upon initial contact, but the dopamine released takes time to elicit an effect on motility.

In contrast, when observing motility that marks the initial slowdown upon encountering the food source (Fig 1B, green box) we observed that in well-fed animals, all biogenic amine mutants reduced their locomotory rate upon entering the food source. This consistent rate of slowdown occurs in all mutants despite the 5-HT deficient mutant having a significantly slower locomotory rate during the chemotaxis phase of the assay (Fig 1E). This initial slowing progresses to the basal slowing rate defined 5 minutes after the worms enter the food for all mutants except DA deficient worms (Fig 1B, teal box).

Having identified a reduced core motility phenotype in the well-fed 5-HT deficient mutant as well as the loss of a food entry slowing phenotype (Fig 1B), we wanted to compare these to the established findings in food deprived worms. This was to ensure we were able to reproduce the behaviour of food entry slowing previously described when investigated in the context of the integrated motility assay format used in this study as described (Iwanir et al., 2016).

Investigation of well-fed and food deprived worms highlights a distinct role for 5-HT between these two contexts (Fig 1A). In well-fed worms, the *tph-1* ( *mg280* ) mutants had a significantly slower baseline locomotory rate (Fig 1B) during chemotaxis. Additional measurements in a bacteria free plate showed that this reflected the role of 5-HT in core locomotory behaviour (Fig 1D) as the 5HT deficient worms when placed onto a no food plate had a significantly slowed spontaneous movement. This reduced core locomotion is not observed in cohorts of N2 and other biogenic amine mutant strains that have undergone prior food deprivation. Thus, based upon the locomotory rate delta during well-fed chemotaxis, which is lost during chemotaxis when food deprived, 5-HT has a discrete effect not seen in the other biogenic amine mutants. This suggests, differential roles for 5-HT in well-fed and food deprived animals in which 5-HT selectively signals to promote locomotion in well-fed animals that is manifest during chemotaxis. However, other neurotransmitters control locomotory rates during chemotaxis in food deprived animals. One potential explanation is 5-HT modulates and sensitises the circuits associated with food entry slowing in a context dependent manner.

In conclusion, the behaviour of basal slowing appears to require an extended time course of dopamine signalling which if perturbed leads to an increase in locomotion after 5 minutes of being on a bacterial lawn. This behaviour is only present in well-fed animals and was unchanged in any of the other biogenic amine mutants suggesting dopamine is the only biogenic amine required for it to function. Secondly, the food entry slowing is context dependent as the food entry slowing phenotype is lost in well-fed 5-HT deficient worms. This could be caused by a sensitisation of the locomotory circuits being governed by 5-HT leading to lower core locomotory rates and increased slowing upon food entry.

## Methods

***C. elegans* husbandry**

All strains utilised were maintained on *E. coli* ( OP50 ) lawns according to standard procedures (Brenner 1974). Strains N2 , *cat-2 ( e1112 )* , *tph-1* ( *mg280* ) *,* *tdc-1* ( *n3419* ) and *tbh-1* ( *n3247* ) were obtained from the CGC, which is funded by NIH Office of Research Infrastructure Programs (P40 OD010440).

**Food Interaction Assay**

Performed as previously described (Sawin et al., 2000)with some modifications. Assay plates were seeded with OP50 24 hours prior and placed at 37ºC 24 hours prior to the assay. Synchronised animal populations were studied at L4+1 day (young adults). Individual animals were picked onto an unseeded NGM plate and allowed to roam for 5 minutes. The cleaned animals were then transferred to the bacteria free centre of the assay plate surrounded by a ring of *E. coli* ( OP50 ). Animals locomotory rate were scored by counting body bends in 20 seconds. Longitudinal analysis involved making video recordings of the assay and measuring the animals locomotory rate by counting body bends over a 20 second period. Measurements were made 20 seconds prior to food entry, immediately after food entry and then every 60 seconds post food entry up to 5 minutes post food entry.

For food deprived animals, they are washed 3 times using sterile M9 buffer and replated onto an unseeded NGM plate and left for 2 hours. These animals were then transferred onto the assay plate as described above.

**Statistics**

Statistics performed with GraphPad Prism 10 and unless otherwise specified analysed using a 2-Way ANOVA with Dunnett's multiple comparisons.

## Reagents

**Table:** 

Strain	Genotype	Available From
N2	*Caenorhabditis elegans*	CGC
CB1112	*cat-2 ( e1112 ) *	CGC
MT15434	*tph-1 ( mg280 ) *	CGC
MT13113	*tdc-1 ( n3419 ) *	CGC
MT9455	*tbh-1 ( n3247 ) *	CGC
